# A comparative study of PD-L1 immunohistochemical assays with four reliable antibodies in thymic carcinoma

**DOI:** 10.18632/oncotarget.24075

**Published:** 2018-01-08

**Authors:** Tadashi Sakane, Takayuki Murase, Katsuhiro Okuda, Hisashi Takino, Ayako Masaki, Risa Oda, Takuya Watanabe, Osamu Kawano, Hiroshi Haneda, Satoru Moriyama, Yushi Saito, Takeshi Yamada, Ryoichi Nakanishi, Hiroshi Inagaki

**Affiliations:** ^1^ Department of Oncology, Immunology and Surgery, Nagoya City University Graduate School of Medical Sciences, Nagoya 467-8601, Japan; ^2^ Department of Pathology and Molecular Diagnostics, Nagoya City University Graduate School of Medical Sciences, Nagoya 467-8601, Japan; ^3^ Department of Chest Surgery, Toyota Memorial Hospital, Toyota 471-8513, Japan; ^4^ Department of Thoracic Surgery, Kariya Toyota General Hospital, Kariya 448-8505, Japan

**Keywords:** programmed death 1 (PD-1), programmed death ligand 1 (PD-L1), thymic carcinoma, squamous cell carcinoma, immunohistochemistry

## Abstract

Currently, four immunohistochemical assays are registered with the US Food and Drug Administration to detect the expression of PD-L1. We investigated the PD-L1 expression in thymic carcinomas using these four diagnostic assays. The cases of 53 patients were reviewed and their specimens were subjected to four PD-L1 assays with different antibodies (SP142, SP263, 22C3, and 28-8). The PD-L1 expression in tumor cells (TCs) and immune cells (ICs) was evaluated. In TCs, the four assays showed similar scores in each case. Histopathologically, high TC scores were observed in squamous cell carcinomas (SqCCs). Meanwhile, there were no significant relationships among the IC scores in the four assays. In SqCCs, the high expression of PD-L1 (defined as ≥50% TC score) in TCs tended to be associated with early stage cancer. The patients with high expression levels of PD-L1 tended to show longer overall survival in the 22C3 assays (p=0.0200). In thymic carcinomas, the staining pattern showed high concordance among the four assays when TCs – rather than ICs – were stained. High PD-L1 positivity in TCs, especially in SqCCs, indicated that PD-1/PD-L1 targeted therapy may be a promising therapeutic approach.

## INTRODUCTION

There is currently no standardized treatment for thymic epithelial tumors because of their low incidence, histological heterogeneity, and unknown molecular pathogenesis [1–3]. In particular, the outcome of thymic carcinoma is often dismal due to the limited response to chemotherapy and the high incidence of distant metastasis [1, 4, 5]. Complete surgical resection is now considered to be the optimum treatment for thymic carcinoma. However, surgery cannot be indicated in some cases because tumors often invade the surrounding organs, such as the heart, nerves, bronchi, and large vessels [3, 4, 6].

Recently, immunotherapy targeting programmed death 1 (PD-1; PDCD1)/programmed death ligand 1 (PD-L1; CD274) has been shown to be clinically effective and thus represents a promising therapeutic alternative in some oncologic cases [7–9]. The binding of PD-1 to its ligand results in the activation of the inhibitory kinases involved in T-cell proliferation, adhesion, and cytokine production/secretion via phosphatase SHP2.2 [7–11]. Several therapeutic agents have been developed to block the PD-1/PD-L1 interaction. The KEYNOTE-010, CheckMate-057 and KEYNOTE-001 studies showed the clinical activity of PD-1 targeted therapies in non-small cell lung cancer (NSCLC) patients and demonstrated that tumors with the high expression of PD-L1 showed an improved response in comparison to tumors with the low (or no) expression of PD-L1 [12–14]. Thus, the expression of PD-L1 is used as a predictive marker or an indication for anti-PD-1/PD-L1 treatment. On the other hand, the association with the patient's prognosis should also be noted. In several reports on different malignancies, the expression of PD-L1 was shown to be associated with a poor prognosis and/or more aggressive disease [7, 9, 15, 16]. A meta-analysis of six studies including 1157 patients with NSCLC revealed that the expression of PD-L1 was associated with poor differentiation of tumors and poor overall survival (OS) [17]. Meanwhile, a few reports have shown that the expression of PD-L1 is correlated with a better prognosis or has no prognostic significance [7, 9, 15]. The prognostic implications of PD-L1 are therefore still uncertain.

Currently, three agents (pembrolizumab [Keytruda, Merck, Kenilworth, NJ, USA], nivolumab [Opdivo, Bristol-Myers Squibb, New York, NY, USA], and atezolizumab [Tecentriq, Genentech/Roche, South San Francisco, CA, USA]) have been approved by the U.S. Food and Drug Administration (FDA) for the treatment of PD-L1-positive NSCLC. Meanwhile, durvalumab (Imfinzi, AstraZeneca, London, United Kingdom) is still under clinical development for use in NSCLC. Several companies have developed different primary antibodies, which have been used to detect PD-L1 proteins in immunohistochemical analyses; these use different staining protocols, scoring algorithms, and threshold criteria. Each FDA-approved agent has its corresponding immunohistochemical assay as a companion or complementary diagnostic test; thus, there is currently a one drug–one diagnostic test co-development approach. Four studies have been performed to compare the companion diagnostic tests for NSCLC, with the aim of better understanding the similarities and differences among the four assays [18–21].

PD-1/PD-L1 targeted therapy has not yet been established for thymic carcinoma. However, the comparison of different assays is essential for selecting appropriate therapies, for achieving a satisfactory clinical outcome in cases of thymic carcinoma, and for promoting the appropriate arrangement of PD-L1 assays in clinical studies. Thus, it is necessary to set up a standard highly reproducible assessment for PD-L1 immunostaining because a scoring system affects the initiation of treatment. The aim of this study is to establish a highly reproducible standard assessment for each companion or complementary PD-L1 antibody in thymic carcinoma and to elucidate the association between the expression of PD-L1 and the clinicopathological features.

## RESULTS

### The clinical and pathological findings

Patients’ characteristics are shown in Table 1. The study population included 32 male patients and 21 female patients (median age, 61 years; range 29–84 years). The tissue types included squamous cell carcinoma (SqCC; n=41, 77.4%), adenocarcinoma (n=4, 7.5%), lymphoepithelioma-like carcinoma (LEC; n=2, 3.8%), carcinoid (n=5, 9.4%), and large cell neuroendocrine carcinoma (n=1, 1.9%). Surgery was performed in 39 patients (73.6%) (surgical cases); complete resection was achieved in 37 of these patients. In 14 patients who did not undergo surgery, the tissues were obtained from biopsy (non-surgical cases). As a matter of course, non-surgical cases tended to be advanced cases. Chemotherapy and/or radiotherapy was administered to most patients for whom surgical resection was not indicated. The median follow-up period in all cases was 38 months (range, 2–166 months). Normal thymic tissues (apart from the tumor) were obtained from five thymoma patients (WHO type A, n=2; and B1, n=3) without autoimmune disease such as myasthenia gravis, as non-neoplastic controls.

**Table 1:** The clinical and pathological findings in the cases of thymic carcinoma

Factor		All cases (n=53)		Surgical cases (n=39)		Non-surgical cases (n=14)	
		Value	%	Value	%	Value	%
Sex	Male	32	60.4	22	56.4	10	71.4
	Female	21	39.6	17	43.6	4	28.6
Age years	median	61		60		64	
	range	29-84		29-84		30-78	
Tumor size (mm)	mean	54.7		52.8		60	
	range	16-120		16-120		31-100	
WHO stage	I	4	7.5	4	10.3	0	0
	II	9	17	9	23.1	0	0
	III	19	35.8	16	41	3	21.4
	IV	21	39.6	10	25.6	11	78.6
Masaoka-Koga stage	I	4	7.5	4	10.3	0	0
	II	9	17	9	23.1	0	0
	III	11	20.8	8	20.5	3	21.4
	IVa	6	11.3	4	10.3	2	14.3
	IVb	23	43.4	14	35.9	9	64.3
Histological subtype	Squamous cell carcinoma	41	77.4	28	71.8	13	92.9
	Adenocarcinoma	4	7.5	4	10.3	0	0
	Lymphoepithelioma-like carcinoma	2	3.8	2	5.1	0	0
	Carcinoid	5	9.4	4	10.3	1	7.1
	Large cell neuroendocrine carcinoma	1	1.9	1	2.6	0	0
Treatment	Surgery	39	73.6	39	100	0	0
	Chemoradiotherapy	9	17	0	0	9	64.3
	Chemotherapy	4	7.5	0	0	4	28.6
	Radiotherapy	0	0	0	0	0	0
	Best supportive care	1	1.9	0	0	1	7.1
Complete resection		37	69.8	37	94.9	0	0

### The immunohistochemical findings

**Figure 1:**
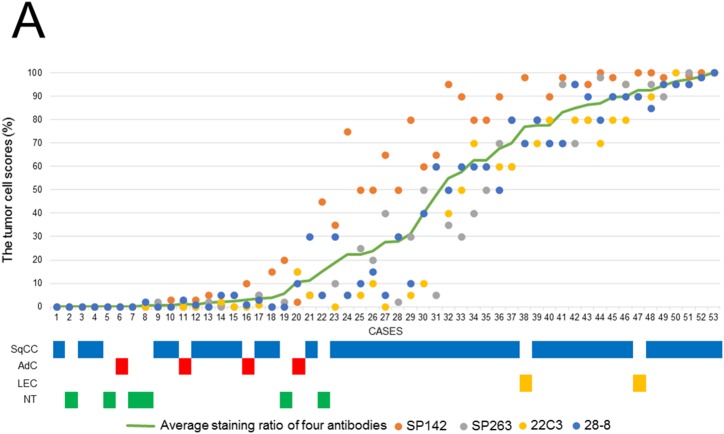

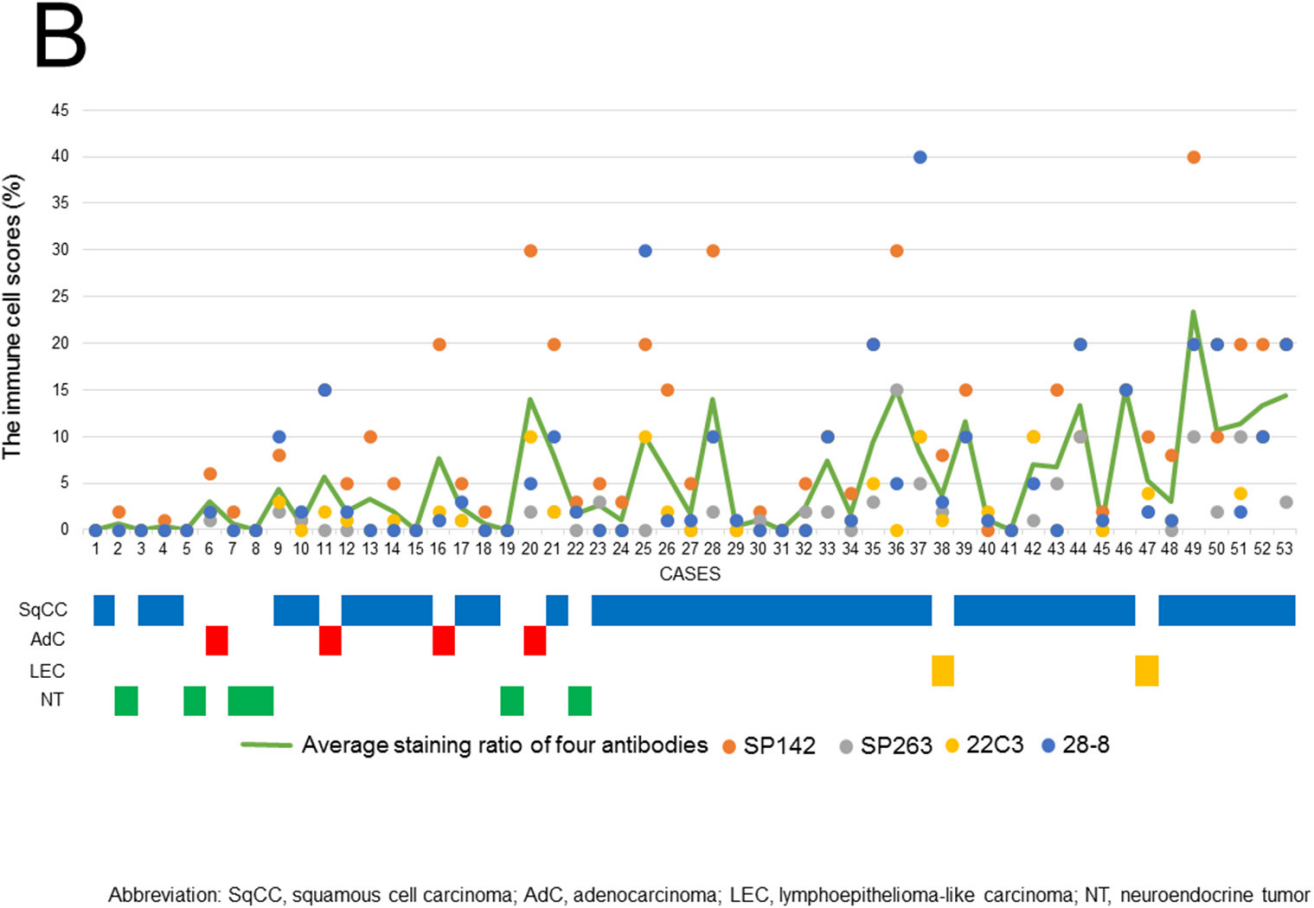
**(A)** The TC scores (the ratio of positive TCs in all carcinoma cells) for the four PD-L1 antibodies (SP142, SP263, 22C3, and 28-8) in each thymic carcinoma patient and the histological subtypes of each patient. **(B)** The IC scores (the ratio of the area covered by stained ICs in the tumor area) for the four PD-L1 antibodies (SP142, SP263, 22C3, and 28-8) in each thymic carcinoma patient and the histological subtypes of each patient.

**Figure 2:**
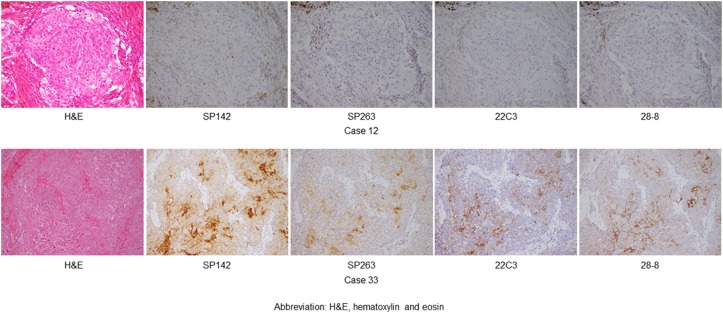
A hematoxylin and eosin-stained specimen and the representative PD-L1 expression in thymic carcinoma (magnification, ×200), as determined by the four assays (SP142, SP263, 22C3, and 28-8) Case 12 shows no or low score in TCs and ICs, while the case 33 shows mid or high score in TCs and ICs.

**Figure 3:**
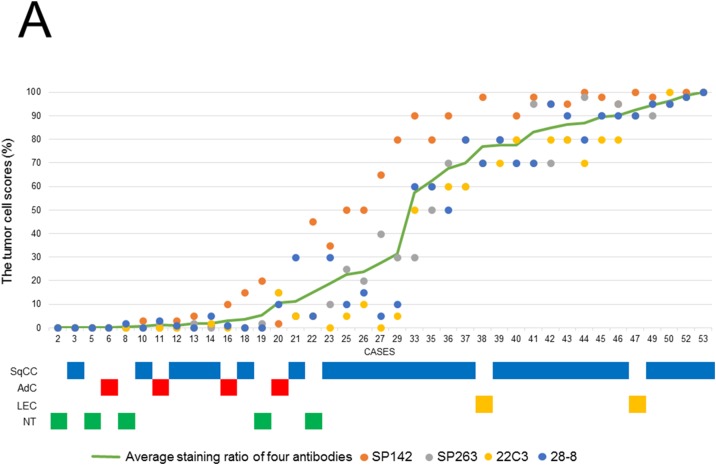

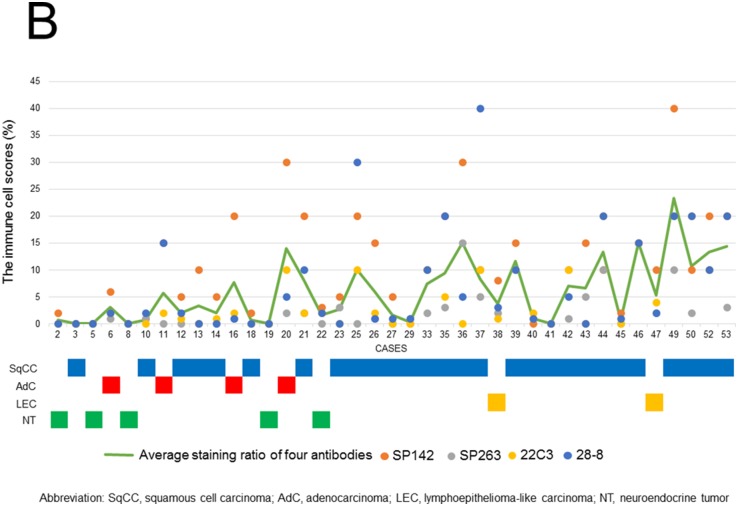
**(A)** The TC scores (the ratio of positive TCs in all carcinoma cells) for the four PD-L1 antibodies (SP142, SP263, 22C3, and 28-8) in each surgical case and the histological subtypes of each case. **(B)** The IC scores (the ratio of the area covered by stained ICs in the tumor area) for the four PD-L1 antibodies (SP142, SP263, 22C3, and 28-8) in each surgical case and the histological subtypes of each case.

**Figure 4:**
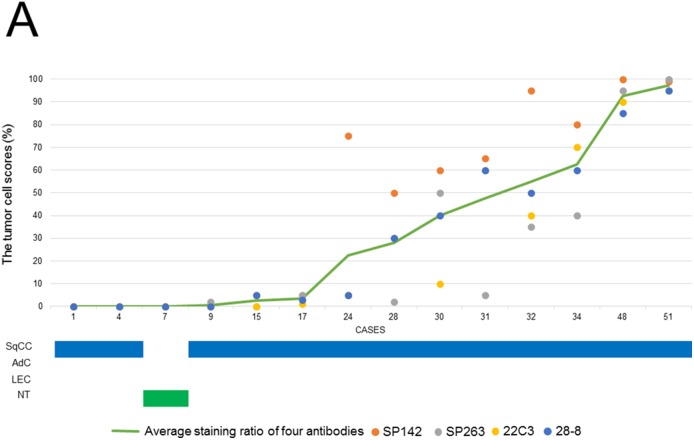

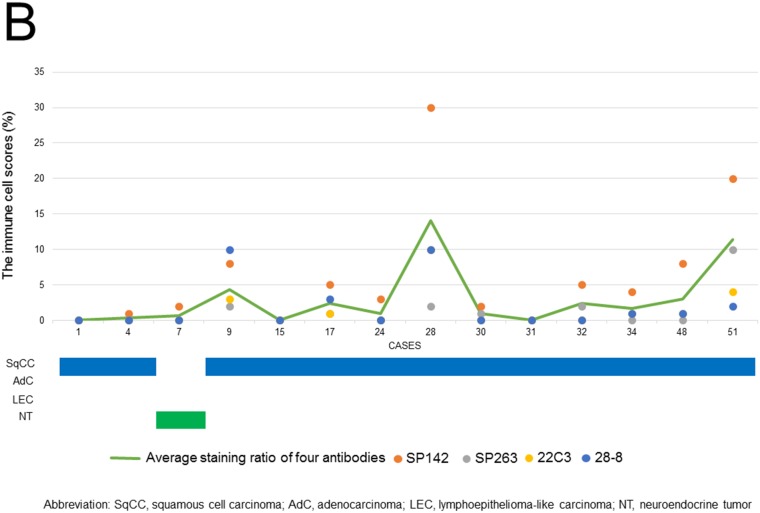
**(A)** The TC scores (the ratio of positive TCs in all carcinoma cells) for the four PD-L1 antibodies (SP142, SP263, 22C3, and 28-8) in each non-surgical case and the histological subtypes of each case. **(B)** The IC scores (the ratio of the area covered by stained ICs in the tumor area) for the four PD-L1 antibodies (SP142, SP263, 22C3, and 28-8) in each non-surgical case and the histological subtypes of each case.

In the 5 thymic control cases, the percentage of PD-L1 positive cells ranged from 0 to 25% in the four assays; the SP142 assay showed a slightly higher expression rate than the other three assays (Supplementary Figure 1). Figure 1A and 1B show the scores of tumor cells (TCs) and immune cells (ICs) in thymic carcinomas and neuroendocrine tumors. In TCs, the four assays showed similar scores in each case, and a higher TC score was detected in one clone (SP142) by the four assays. Meanwhile, there were no significant relationships among the IC scores in the four assays (Figure 1B). As a representative example, hematoxylin and eosin (H&E)-stained and PD-L1-stained specimens with the four antibodies of case 12 and 33 are shown in Figure 2. Histologically, a higher TC score was detected in SqCCs and LECs, while lower TC scores were detected in adenocarcinomas, and neuroendocrine tumors (Figure 1A). Next, the TC and IC scores were examined according to the method in which the specimen was obtained (surgically resected or biopsy) (Figures 3 and 4). The TC and IC scores of the surgical and non-surgical cases were compared for each of the four antibodies. No significant difference was observed in the TC scores of the two groups (p=0.2946-0.6123). With regard to the IC score, although the surgical specimens showed higher scores than the biopsy specimens in the 28-8 assay (p=0.0336), no significant difference was found between the two groups in the other three assays (p=0.0830-0.1654).

**Figure 5:**
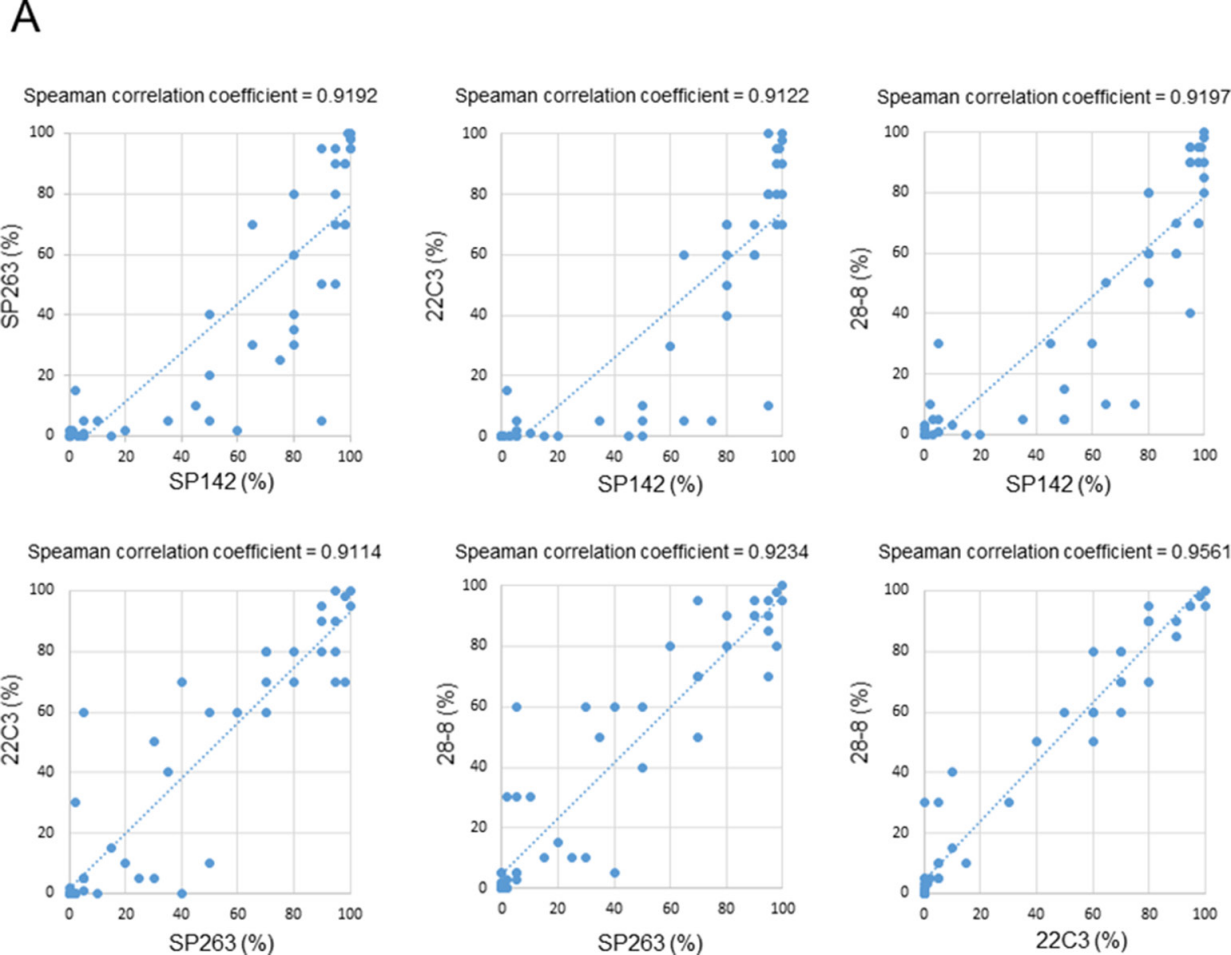

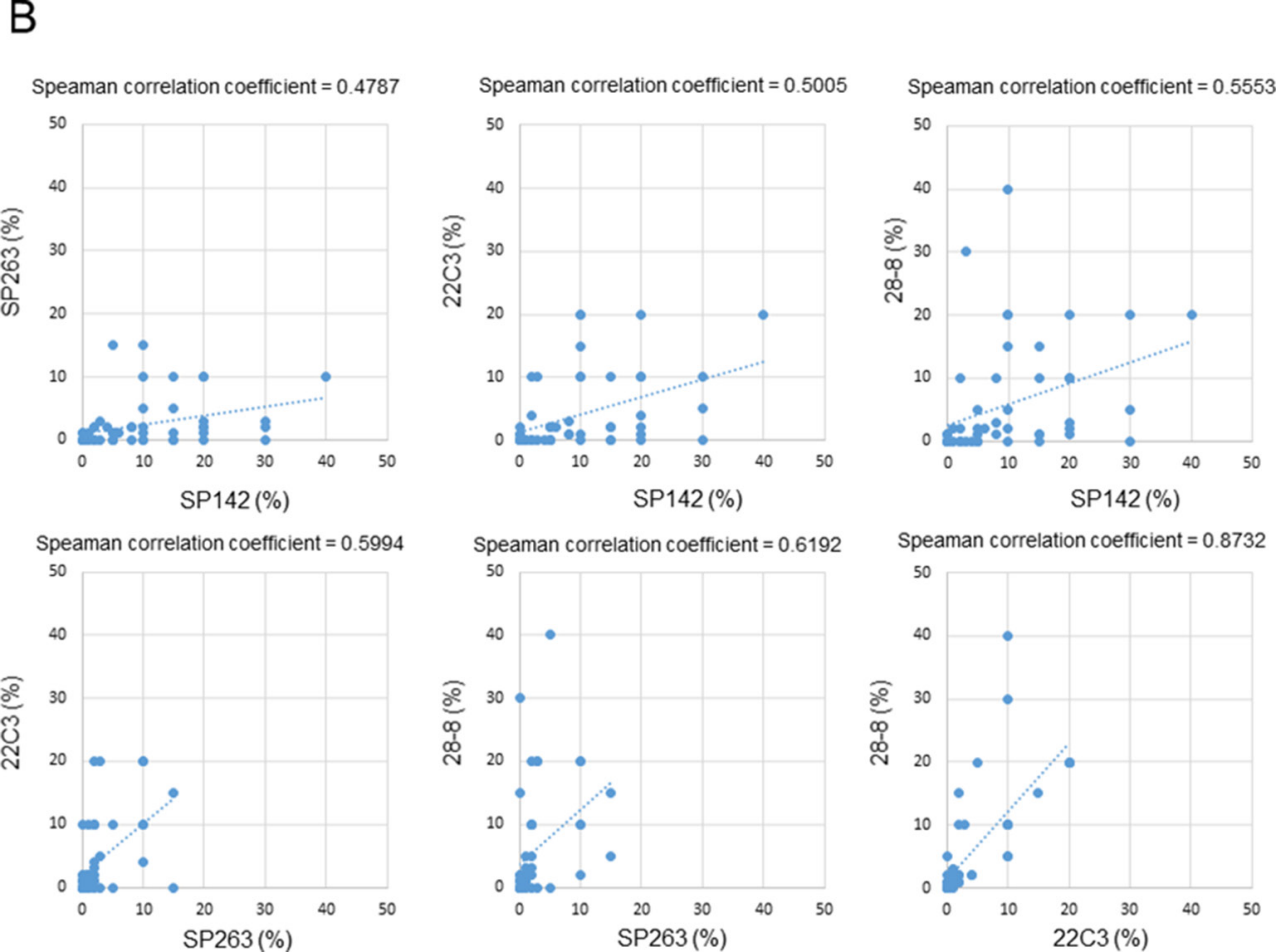
**(A)** Pairwise analyses of the TC scores in thymic carcinomas for the four PD-L1 antibodies (SP142, SP263, 22C3, and 28-8). **(B)** Pairwise analyses of the IC scores in thymic carcinomas for the four PD-L1 antibodies (SP142, SP263, 22C3, and 28-8).

The distribution of the PD-L1 expression, as detected by the four antibodies, is shown in Supplementary Figure 2. Approximately 50% of the cases expressed PD-L1 in more than 50% of all TCs in four assays. Figure 5 shows the TC and IC scores with each antibody for all of the possible pairwise comparisons between the four assays. The 45-degree regression line indicates the correlation with the TC and IC scores. Each plot compares the assays indicated in the x- and y-axis labels. In the TC scores, the Spearman's rank correlation coefficients were all >0.9, indicating high levels of correlation among the four assays. In the IC scores, the Spearman's rank correlation coefficients were all >0.4, but <0.9, indicating moderate levels of association among the four assays.

### PD-L1 expression using the validated cutoffs for each assay

**Figure 6:**
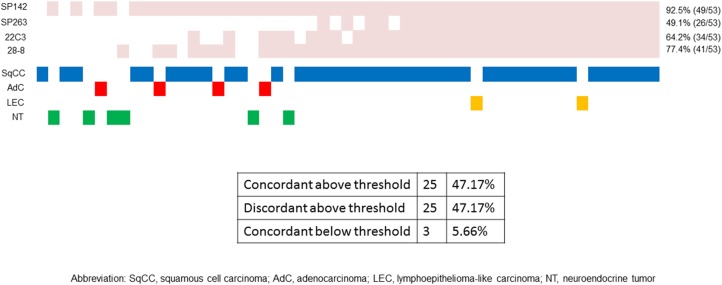
The heat map for each case based on the PD-L1 expression The cases in which the values were below the validated PD-L1 cutoffs are shown in white. The cases in which the values were greater than or equal to the validated PD-L1 cutoffs are shown in pink. The concordance or discordance among the clinical PD-L1 expression was evaluated for each case. The histological subtypes were also evaluated.

The PD-L1 expression status (above or below the assay threshold) in all samples was compared using the cutoffs applied to NSCLC. We determined the concordance among the four assays when classifying cases according to the assay and the selected cutoff combination and specified each drug and assay combination. The heat map in Figure 6 illustrates, on a case-by-case basis, the cases in which TCs expressed PD-L1 at levels above or below the validated cutoffs for each assay. The cases in which the values were below the validated PD-L1 cutoffs are shown in white, while those that were greater than or equal to the validated PD-L1 cutoff values are shown in pink. The SP142 assay showed that 49 of the 53 cases (92.5%) were greater than or equal to the cutoff value; the SP263 assay showed 26 of 53 cases (49.1%); the 22C3 assay showed 34 of 53 cases (64.2%); and the 28-8 assay showed 41 of 53 cases (77.4%). Twenty-five of 53 cases (47.2%) had values that were greater than or equal to the cutoff values utilized by all of the assays, meaning that clinical PD-L1 positivity would be concordant regardless of the assays that were used. Twenty-five cases (47.2%) showed discordance between the clinical PD-L1 expression levels. Three cases (5.7%) were found to be below the cutoff values, irrespective of the assay that was used. More consistent results were observed in the cases with higher TC scores than in those with lower scores. This means that concordance at levels that were greater than or equal to the threshold value was especially seen in SqCCs and LECs. On the contrary, discordance at levels above the threshold value was most frequently seen in adenocarcinomas and neuroendocrine tumors.

### Statistical analysis

**Figure 7:**
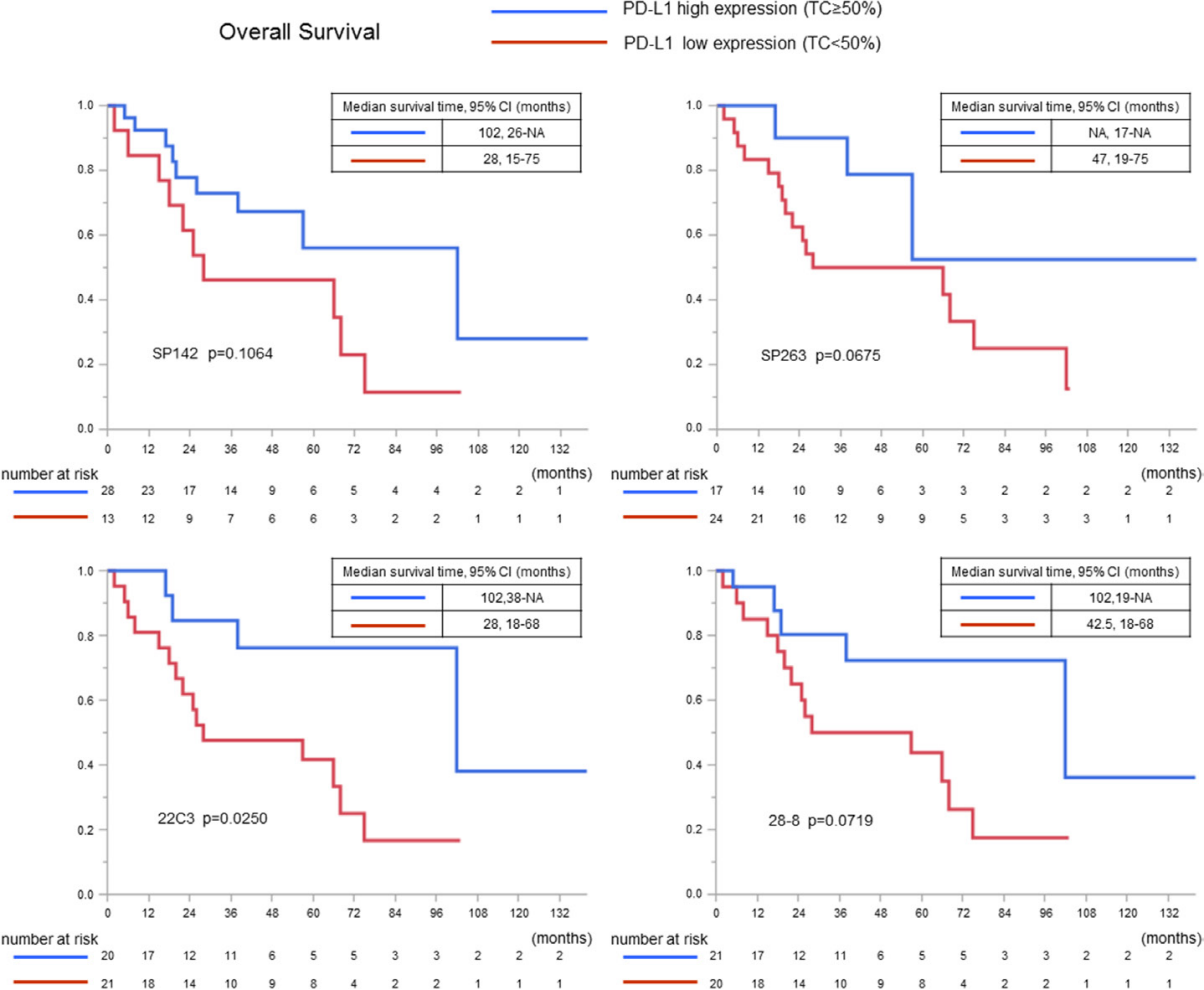
The overall survival curves in all squamous cell carcinoma cases according to the PD-L1 expression (using a cutoff value of 50%)

**Figure 8:**
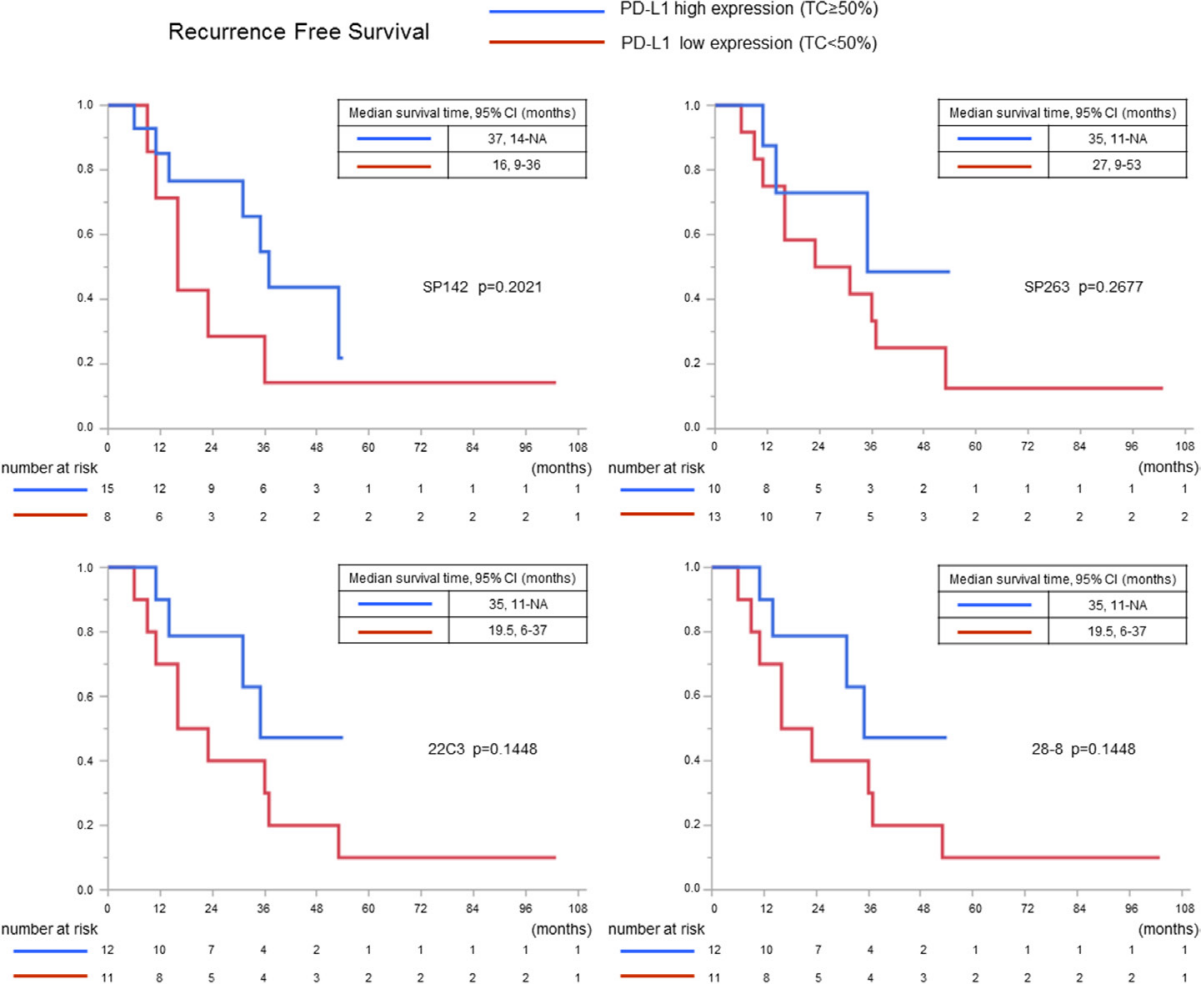
The recurrence-free survival curves in squamous cell carcinoma cases in which complete resection was performed according to the PD-L1 expression (using a cutoff value of 50%)

The clinicopathological features in all thymic carcinoma patients and the PD-L1 expression are shown in Table 2. Three assays (SP142, SP263, and 22C3) showed that patients who expressed PD-L1 were more likely to exhibit SqCCs (p=0.0053-0.0194). In only SP142 assay the association remained significant after the Bonferroni correction. The expression of PD-L1 was not correlated with sex (p=0.7822-1.0000), age (p=0.1662-1.0000), WHO stage (0.1744-1.0000), Masaoka-Koga stage (0.1024-1.0000), primary tumor maximum diameter (p=0.0690-0.5433), or curability (p=0.2444-1.0000). When we examined the relationship between clinicopathologic features and the PD-L1 expression only in surgical cases, similar results were obtained (Supplementary Table 1). Next we used 50% cutoff values based on the KEYNOTE-010, KEYNOTE-001 and POPLAR studies [12, 14, 22]. The correlation between the clinicopathological features and the high expression of PD-L1 (defined by ≥50% PD-L1-positive TCs) in each assay was analyzed in the cases of SqCC (Table 3), that occupies the majority of the histopathological findings of thymic carcinomas as well as in this study. Three assays with clones SP263, 22C3, and 28-8 showed an association between the high expression of PD-L1 and an early stage according to the WHO and Masaoka-Koga staging systems (p=0.0205-0.0486 and p=0.0205-0.0486, respectively). After the application of the Bonferroni correction, however, all associations lost significance. The OS curve of patients with SqCC and the recurrence-free survival (RFS) curve after complete surgical resection for patients with SqCC—after the patients were stratified according to the expression of PD-L1— are shown in Figures 7 and 8. The OS was significantly longer in patients in whom ≥50% of the TCs were PD-L1-positive in 22C3 assay (p=0.0250), whereas the OS tended to be longer in patients in whom ≥50% of the TCs were PD-L1-positive in the three assays (p=0.1064 with clone SP142, p=0.0675 with clone SP263, and p=0.0719 with clone 28-8). The prognostic value of each clinicopathological factor was evaluated. A univariate analysis revealed that the high expression of PD-L1 (using a cut-off value of 50%) in TCs in the 22C3 assay was associated with greater OS (p=0.0200); no significance was found in a multivariate analysis with multicollinearity eliminated (p=0.3007) (Table 4). The PD-L1 expression status was not an independent predictor of RFS (Supplementary Table 2). The details of the multicollinearity in the multivariate Cox regression analysis for OS and PFS are shown in Supplementary Tables 3 and 4.

**Table 2:** The statistical analysis of all histological subtypes of thymic carcinoma

Factor		SP142			SP263			22C3			28-8		
		TC≧1%	TC<1%	p value	TC≧25%	TC<25%	p value	TC≧1%	TC<1%	p value	TC≧1%	TC<1%	p value
	Total cases	43	10		26	27		34	19		41	12	
Sex	Male	26	6	1.0000	15	17	0.7822	21	11	1.0000	25	7	1.0000
	Female	17	4		11	10		13	8		16	5	
Age years	≧60	23	8	0.1662	15	16	1.0000	20	11	1.0000	22	9	0.3182
	<60	20	2		11	11		14	8		19	3	
WHO stage	Stage I/II	10	2	1.0000	8	4	0.2021	10	2	0.1744	11	1	0.2565
	Stage III/IV	33	8		18	23		24	17		30	11	
Masaoka-Koga stage	Stage I/II	11	2	1.0000	9	4	0.1188	11	2	0.1024	12	1	0.2529
	Stage III/IV	32	8		17	23		23	17		29	11	
Tumor size (mm)	≧50	22	6	0.2268	15	13	0.5433	18	10	0.0962	22	6	0.0690
	<50	16	1		11	6		15	2		17	0	
Curability	Complete resection	31	8	1.0000	21	18	0.2444	24	15	0.7463	30	9	1.0000
	Imcomplete resection/biopsy	12	2		5	9		10	4		11	3	
Histological subtype	Squamous cell carcinoma	37	4	0.0053	24	17	0.0194	30	11	0.0175	34	7	0.1143
	Other type carcinoma	6	6		2	10		4	8		7	5	

Abbreviation: TC, tumor cells.

**Table 3:** The statistical analysis of the cases of thymic squamous cell carcinoma using a cutoff value of 50%

Factor		SP142			SP263			22C3			28-8		
		TC≧50%	TC<50%	p value	TC≧50%	TC<50%	p value	TC≧50%	TC<50%	p value	TC≧50%	TC<50%	p value
	Total cases	28	13		17	24		20	21		21	20	
Sex	Male	17	8	1.0000	11	14	0.7533	14	11	0.3408	14	11	0.5303
	Female	11	5		6	10		6	10		7	9	
Age	≧60	15	8	0.7417	10	13	1.0000	12	11	0.7557	12	11	1.0000
	<60	13	5		7	11		8	10		9	9	
WHO stage	Stage I/II	7	1	0.3983	6	2	0.0486	7	1	0.0205	7	1	0.0448
	Stage III/IV	21	12		11	22		13	20		14	19	
Masaoka-Koga stage	Stage I/II	7	1	0.3983	6	2	0.0486	7	1	0.0205	7	1	0.0448
	Stage III/IV	21	12		11	22		13	20		14	19	
Tumor size (mm)	≧50	17	5	0.7115	9	13	0.4955	10	12	0.1760	11	11	0.3021
	<50	10	4		8	6		10	4		10	4	
Curability	Complete resection	17	8	1.0000	12	13	0.3444	14	11	0.3408	14	11	0.5303
	Imcomplete resection/biopsy	11	5		5	11		6	10		7	9	

Abbreviation: TC, tumor cells.

**Table 4:** The results of the univariate and multivariate analyses of prognostic factors affecting the OS in patients with thymic squamous cell carcinoma

Factor		Overall survival					
		univariate analysis			multivariate analysis		
		HR	95% CI	p value	HR	95% CI	p value
Sex	Male	0.974	0.392-2.529	0.9548			
	Female						
Age years	≧60	0.563	0.217-1.398	0.2155			
	<60						
WHO stage	Stage I/II	0.233	0.013-1.140	0.0778	0.437	0.023-2.581	0.4065
	Stage III/IV						
Masaoka-Koga stage	Stage I/II	0.233	0.013-1.140	0.0778	NA	NA	NA
	Stage III/IV						
Tumor size (mm)	≧50	1.681	0.494-7.633	0.4227			
	<50						
Curability	Complete resection	0.645	0.254-1.617	0.3451			
	Imcomplete resection/Biopsy						
SP142 1% cutoff		1.174	0.371-5.220	0.8031			
SP263 25% cutoff		0.396	0.138-1.011	0.0528	0.718	0.162-2.283	0.5993
22C3 1% cutoff		0.521	0.208-1.324	0.1665			
28-8 1% cutoff		0.676	0.260-1.974	0.4519			
SP142 50% cutoff		0.482	0.191-1.201	0.1156			
SP263 50% cutoff		0.333	0.077-1.005	0.0512	0.888	0.136-5.906	0.8992
22C3 50% cutoff		0.301	0.085-0.837	0.0200	0.481	0.096-1.813	0.3007
28-8 50% cutoff		0.400	0.128-1.057	0.0652			

Abbreviation: OS, overall survival; HR, hazard ratio; CI, confidence interval.

## DISCUSSION

PD-L1 is a transmembranous protein that downregulates immune responses by binding to its two receptors, PD-1 and B7.1 [8, 9, 23]. PD-L1 is broadly expressed on various immune or non-immune cell types, such as T-cells, B-cells, macrophages, regulatory T-cells, and dendritic cells or various tumor cells and virus-infected cells [7–9, 11].

Six previous studies have addressed the immunohistochemical expression of PD-L1 in TCs in the context of the patients with thymic carcinomas. In 2015, Padda et al. evaluated the PD-L1 expression in four patients using clone 15 (Sino Biological, Beijing, China) and reported that the expression of PD-L1 was confirmed in three patients (75.0%) [24]. Katsuya et al., Weissferdt et al. and Inaguma et al., who used clone E1L3N (Cell Signaling Technology, Danvers, MA, USA), reported that the expression of PD-L1 was confirmed in 26 of 37 patients (70.3%), in 14 of 26 patients (53.8%) and in 6 of 16 patients (37.5%), respectively [25–27]. Yokoyama et al., who used clone EPR1161 (Abcam, Cambridge, MA, USA), reported that the high expression of PD-L1 was confirmed in 20 of 25 patients (80.0%) [28]. In 2016, Marchevsky et al. evaluated the PD-L1 expression in eight patients using clone SP142, which we also used in the present study, and they reported that the expression was confirmed in four patients (50.0%) [29]. Padda et al. and Marchevsky et al. also evaluated the PD-L1 expression in non-neoplastic thymic epithelial cells; the latter group reported that in 18 of 20 cases the rate of PD-L1-positive cells was ≤20%. The former group showed no detailed data on the proportion of positive cells. Our results with non-neoplastic thymic epithelial cells were similar to their results [24, 29]. According to our study, which used four antibodies, the PD-L1 protein was positively expressed in 49.1-81.1% of TCs. These rates were relatively similar to the rates reported in six previous studies. Our study also supported the possibility that PD-1/PD-L1 blockade may be a therapeutic alternative for the treatment of thymic carcinoma.

One aspect of the present study that was superior to previous studies: the present study clarified the differences in the expression of PD-L1 according to the tissue type in thymic carcinoma. We found that the expression of PD-L1 in the TCs in patients with SqCCs and LECs was significantly high in comparison to adenocarcinomas and neuroendocrine tumors. Similar results were reported in patients with NSCLC and uterine cervical cancer [30, 31]. Yu et al. and Tsuruoka et al. reported that the frequency of PD-L1 positivity was low in neuroendocrine tumors of the lung, including small cell carcinomas [32, 33]. Interestingly, the LEC patients in our study showed high PD-L1 levels, and a similar tendency was reported in patients with lung LEC [34]. Although the etiology of LECs is unknown, some cases are associated with Epstein-Barr virus [5]. As mentioned above, viral infection and subsequent chronic inflammation might be associated with the expression of PD-L1 in LEC patients.

Several recent studies using different assays compared the expression of PD-L1 in NSCLC and showed the slight superiority of the assay using clone SP263, which showed the highest concordance rates for TC scoring, while 22C3 and 28-8 showed comparable yields; SP142 showed the lowest concordance rates for TCs [18–21]. This study showed the slight superiority of the SP142 assay for TCs scoring. There are some explanations for this discrepancy. First, SP142 and SP263 antibodies bind to the intracellular domain of PD-L1, whereas 22C3 and 28-8 antibodies bind to the extracellular domain [35]. This difference in the binding domains alters the sensitivity and specificity of the detection assay. Second, the type, processing, storage, and amount of tissue might affect the ability to detect PD-L1 in the tumor. In this study, approximately one quarter of the samples were obtained from biopsies. Reports have shown that the surgical specimens showed a higher rate of PD-L1 positivity than biopsy specimens, although we could not confirm this in our study [36, 37]. There are also some differences in the prevalence of PD-L1 between fresh frozen tissue and formalin-fixed, paraffin-embedded (FFPE) tissue [26]. Furthermore, Yu et al. reported that slides stained within 90 days had a slightly higher prevalence PD-L1 positivity (24%) in comparison to samples that were stored for ≥90 days (11%) [32]. The denaturant effect of formalin fixation on protein could also compromise antigen staining during immunohistochemistry. The current use of such non-standardized immunohistochemical techniques for measuring the PD-L1 levels in tissue might have some influence on the results. Of course it should be kept in mind that the results of this validation trial for thymic carcinomas are not necessarily similar to the results for NSCLC. Anyway it will be important to develop standardized methods for evaluating PD-L1 by immunohistochemistry.

In IC staining, no significant correlations were observed among the four assays. Tumor-infiltrating lymphocytes (TILs) are measured morphologically, and there are no established thresholds for TILs at present [38]. However, the presence of TILs, a key component of the tumor microenvironment, is a favorable prognostic factor in numerous cancers [23, 39, 40]. In the future, we need to establish a more objective and simple method for evaluating IC staining, and the significance of the expression of PD-L1 in ICs should be analyzed.

Different results regarding the relationship between the PD-L1 expression and the patient's prognosis have been reported in various studies of various carcinomas [7, 9]. In thymic epithelial tumors, including thymic carcinomas, the prognostic implications of PD-L1 are still uncertain. Yokoyama et al. found that thymic carcinoma patients with high PD-L1 expression levels had superior OS in comparison to patients with low PD-L1 expression levels [28]. However, they found no correlation between the PD-L1 expression and the Masaoka-Koga stage [28]. On the other hand, Weissferdt et al. detected no association between the expression of PD-L1 and stage or OS [26]. Katsuya et al. also detected no association between the expression of PD-L1 and OS [25]. In this study, there was a correlation trend between the PD-L1 expression and stage (WHO and Masaoka-Koga), when we used a 50% cutoff value with SP263, 22C3, and 28-8. In the analysis of the association between PD-L1 expression and the prognosis, our results were similar to those of Yokoyama et al [28]. This discrepancy regarding the associations between the PD-L1 expression and the prognosis or the characteristics of the disease may be attributable to limited study populations, and differences in antibodies, cutoff values, specimen conditions and pathologists. However, we should consider the fact that thymic epithelial tumors, unlike other tumors, are frequently associated with autoimmune conditions and immune dysfunction, and the PD-1/PD-L1 interaction is critical for thymocyte selection in the normal thymus [3, 4, 41–43]. In the near future, the administration of immune checkpoint inhibitors, including the blockade of the PD-1/PD-L1 pathway, will probably be an effective therapeutic approach for the treatment of thymic epithelial tumors. However, the application to thymic epithelial tumors may not proceed as smoothly as it does with other solid tumors unless we elucidate the interactions between immune checkpoint inhibitors and thymocytes.

A large number of companies and academic laboratories are attempting to develop antibodies to detect PD-L1 as a potential predictive marker for therapies that interfere with the interaction of one or both of the PD-1 ligands and the PD-1 receptor. The best PD-L1 antibody and the appropriate cutoff expression level for determining the PD-L1 expression remain controversial. The cutoff PD-L1 expression levels that were used in this study ranged from 1% to 50%, depending on the previous studies and assays that were used for lung cancer patients. The KEYNOTE-010 study compared pembrolizumab to docetaxel in previously treated patients with NSCLC, using 1% and 50% cutoff values for the expression of PD-L1 [12]. Pembrolizumab showed significant superiority to docetaxel in terms of OS and the objective response rate at both cutoff values, while the median OS tended to be higher in patients with >50% PD-L1 positivity. The POPLAR study, in which atezolizumab was compared to docetaxel in previously treated, advanced, or metastatic NSCLC patients, also showed that the OS increased in association with an increase in the expression of PD-L1 [22]. The clinical significance of 50% cutoff value in TCs is being examined [12, 14, 22]. In the clinical practice, Pembrolizumab is already approved by FDA for first-line treatment of patients with metastatic NSCLC who shows ≥50% PD-L1-positive TCs, with no EGFR or ALK genomic tumor aberrations [44]. On the other hand, the OAK study, which compared atezolizumab to docetaxel in patients with NSCLC, showed that the OS of patients treated with atezolizumab was superior, even in the PD-L1-negative patients [45]. Thus, the cutoff value needs to balance the drive for higher efficacy in patients selected for treatment versus the opportunity to benefit the highest number of patients. The appropriate cutoff PD-L1 value may also depend on the particular therapy that is used and needs to be determined in a robust clinical study. A number of different cutoff PD-L1 values are therefore likely to be applied for selecting different therapies. In this study, the cutoff values for each antibody that were adopted were the same as those used in clinical trials in patients with NSCLC. In patients with thymic carcinoma, the high expression of PD-L1 (defined by ≥50% PD-L1 positivity in TCs, which is also adopted for SP142 and 22C3 antibodies in the previous studies), tended to be more strongly associated with the clinicopathological features and prognosis than the PD-L1 expression when a 1% cutoff value was used. The cutoff values indicating the induction of PD-1/PD-L1 antibodies and for predicting the efficacy of the PD-1/PD-L1 blockade in patients with thymic carcinoma are not necessarily the same as those in patients with lung cancer. In the future, clear cutoff values for thymic carcinomas should be established.

To the best of our knowledge, this is the largest study ever carried out on thymic carcinomas, and it is also the first validation trial conducted on patients except for lung cancer patients using four companion or complementary diagnostic tests. We believe that this research gives us very realistic information that will be useful in the clinical administration of therapeutic drugs. The present study was associated with several limitations. First, the specimens that were used for the immunohistochemical examinations were not all from surgically resected tissues. Second, the auto-stainer that was used for immunohistochemistry for SP142 and SP263 was not the Ventana Benchmark Ultra, which is an optimized platform. Third, despite the fact that the present study included 53 cases of thymic carcinomas and thymic neuroendocrine tumors, which was a relatively large sample size, further prospective studies with more cases and a longer follow-up period are needed. Further studies are required, including prospective analyses of large cohorts and biological analyses to investigate genomic abnormalities or PD-L1 transcriptional levels in thymic carcinomas.

In conclusion, we evaluated the PD-L1 expression in TCs and ICs in patients with thymic carcinomas, with the aim of achieving the harmonization of the four immunohistochemical assays. This study revealed that patients with thymic carcinomas, especially SqCC patients, showed high PD-L1 positivity and tended to have a favorable prognosis. Furthermore, high concordance in the four assays—which showed greater utility in TC staining than in IC staining—was observed. Our results suggest that the PD-1/PD-L1 pathway is a potential immunotherapeutic target in thymic carcinoma.

## MATERIALS AND METHODS

### Patients

We reviewed FFPE tissue specimens from 53 patients with thymic carcinoma or neuroendocrine tumors and five controls of non-neoplastic thymic epithelial cells adjacent to thymomas. All of the specimens were obtained from Nagoya City University Hospital, Toyota Memorial Hospital, and Kariya Toyota General Hospital from 1984 to 2016. There were 39 surgical specimens and 14 needle biopsy specimens. All of the cases were microscopically reviewed and diagnosed by two expert pathologists (TM and HI). No specimens associated with any therapeutic trial or immune checkpoint inhibitor therapy were included in this study. The relevant clinical data were collected from the patients’ medical records. This study was approved by the Institutional Review Boards of the abovementioned institutions and was carried out in accordance with the Declaration of Helsinki.

### Immunohistochemistry

The H&E-stained specimens in each case were microscopically reviewed and each case was pathologically diagnosed according to the 2015 WHO classification [5]. An appropriate FFPE block containing the tumor in each case was selected by reviewing H&E-stained specimens and each FFPE block was sliced into 3-μm-thick tissue sections. The tissue sections were deparaffinized and subjected to immunostaining with the following four anti-PD-L1 antibodies: clone SP142 (Ventana Medical Systems, Tucson, AZ, USA), clone SP263 (Ventana Medical Systems), clone 22C3 (Dako, Carpinteria, CA, USA), and clone 28-8 (Dako). SP142 and SP263 immunostaining was carried out with a Bond-Max autoimmunostainer (Leica Microsystems, Wetzlar, Germany) and a Bond polymer-refine detection kit (Leica Microsystems). 22C3 and 28-8 immunostaining was carried out with a Dako autostainer Link48 system (Dako) and a PD-L1 PharmDx kit (Dako).

### PD-L1 scoring

The positivity of TCs and ICs was assessed by two expert pathologists (TM and HI). TCs in which the membrane was immunostained at any intensity were considered to be positive for PD-L1. The ratios of PD-L1-positive TCs in all of the carcinoma cells were evaluated by microscopic observation (TC scores). Meanwhile, ICs in which the membrane or cytoplasm was immunostained at any intensity were considered to be positive for PD-L1, because the stained membrane and cytoplasm could not be distinguished in lymphocytes due to their small size. Most PD-L1-positive ICs were macrophages and lymphocytes. ICs were quantified by evaluating the ratio of the area covered by stained ICs in the tumor area (IC scores), as described in previous reports [22, 45, 46]. The tumor area was defined as the area occupied by viable TCs and their associated intratumoral and contiguous peritumoral stroma [23]. The necrotic areas were excluded from the scoring area. Although cases with <100 viable TCs were excluded from the present study, all of the examined cases contained >100 TCs. Negative reagent controls were evaluated in each case by confirming the acceptable level of background staining. The cutoff values were settled at TC 1% or IC 1% for SP142, TC 25% for SP263, TC 1% for 22C3 and TC 1% for 28-8; the cutoff positive staining ratio was determined based on the clinical response to anti PD-1/PD-L1 therapy in previous reports [12–14, 22, 45–47].

### Statistical analysis

The correlations between the PD-L1 expression and the clinicopathological features were evaluated by Wilcoxon rank-sum test for continuous variables (the TC and IC scores between surgical cases and non-surgical cases) and by Fisher's exact test for categorical variables (sex, age [<60 years of age or ≥60 years of age], histopathology [squamous cell carcinoma or other types of carcinoma], WHO stage [Stage I, II or III, IV], Masaoka–Koga stage [Stage I, II or III, IV], primary tumor maximum diameter [< 50 mm or ≥50 mm], and curability [complete resection or incomplete resection/biopsy]). Bonferroni correction was used to account for multiple comparisons as appropriate. Spearman's correlation coefficient was calculated to evaluate the correlation regarding PD-L1 positivity in each assay. Survival curves were generated using the Kaplan-Meier method, and the log-rank test was used to assess the statistical significance of differences between groups. A Cox proportional hazards model was used to estimate the hazard ratios and 95% confidence intervals. The prognostic variables identified by a univariate analysis were further analyzed in a multivariate Cox model, where we evaluated the variance inflation factors to assess the multicollinearity for the proposed model. A two-sided p value of <0.05 was considered to indicate a statistically significant difference. The statistical significance level was adjusted for multiple analyses according to Bonferroni correction. All of the statistical analyses in this study were performed using the JMP software program (version 12.0.1, SAS Institute, Tokyo, Japan).

## SUPPLEMENTARY MATERIALS FIGURES AND TABLES



## Abbreviations

PD-1: programmed death 1
PD-L1: programmed death ligand 1
NSCLC: non-small cell lung cancer
OS: overall survival
FDA: the U.S. Food and Drug Administration
SqCC: squamous cell carcinoma
LEC: lymphoepithelioma-like carcinoma
TC: tumor cell
IC: immune cell
H&E: hematoxylin and eosin
RFS: recurrence-free survival
FFPE: formalin-fixed
TILs: Tumor-infiltrating lymphocytes.
