# Supplementary material for: A comparative study of PD-L1 immunohistochemical assays with four reliable antibodies in thymic carcinoma
Source: Oncotarget. 2018 Jan 8;9(6):6993–7009. doi: 10.18632/oncotarget.24075 (PMC5805531; doi:10.18632/oncotarget.24075)
Supplement: Supplementary file 1 [file oncotarget-09-6993-s001.pdf]

A comparative study of PD-L1 immunohistochemical assays with four reliable antibodies in thymic carcinoma

SUPPLEMENTARY MATERIALS

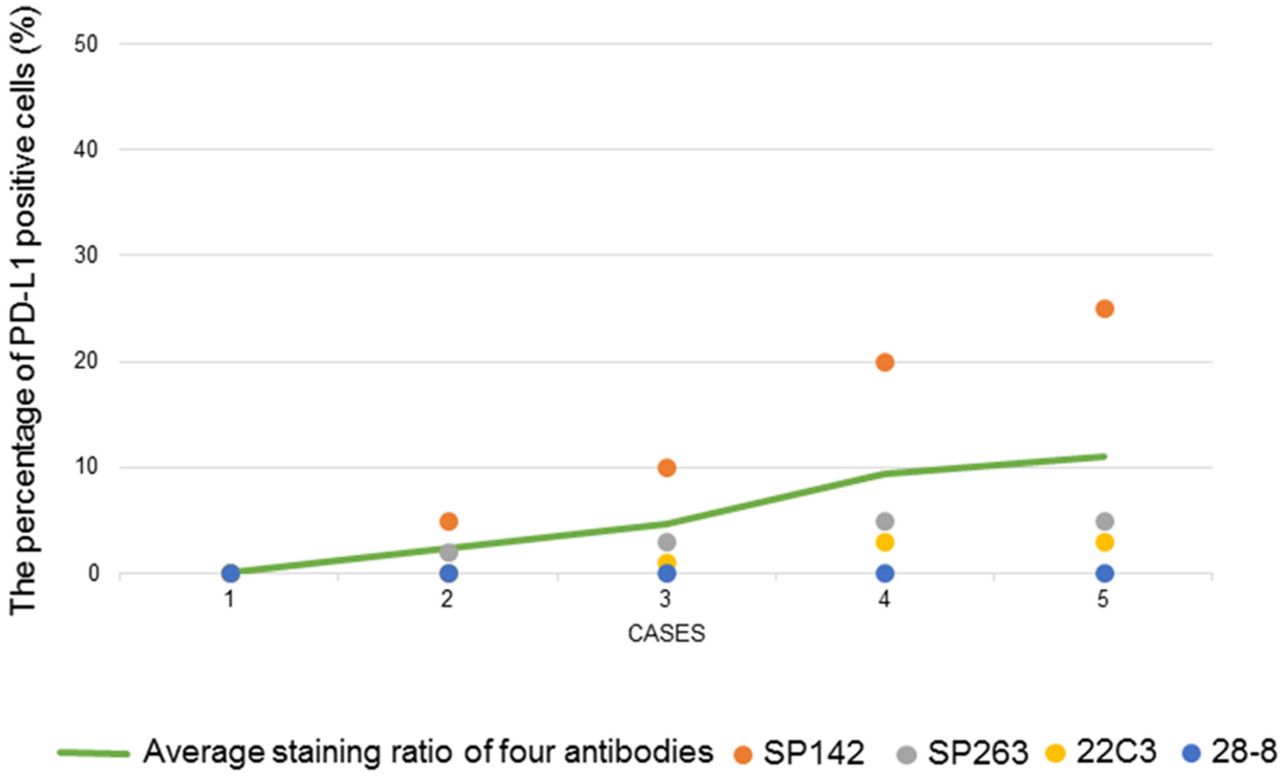

Supplementary Figure 1: The percentage of PD-L1positive thymic epithelial cells in five control cases.

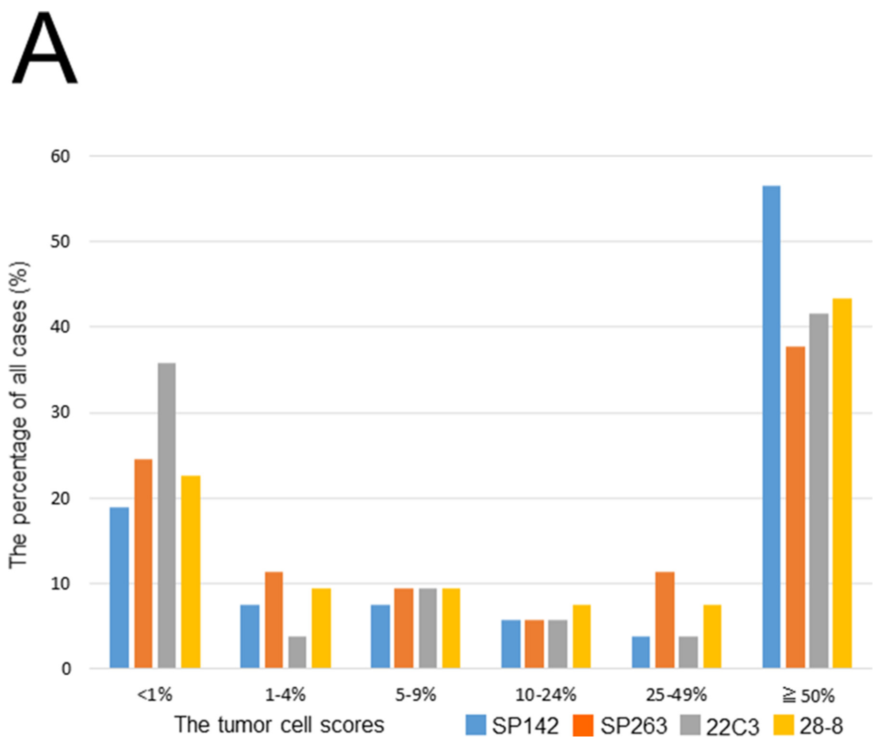

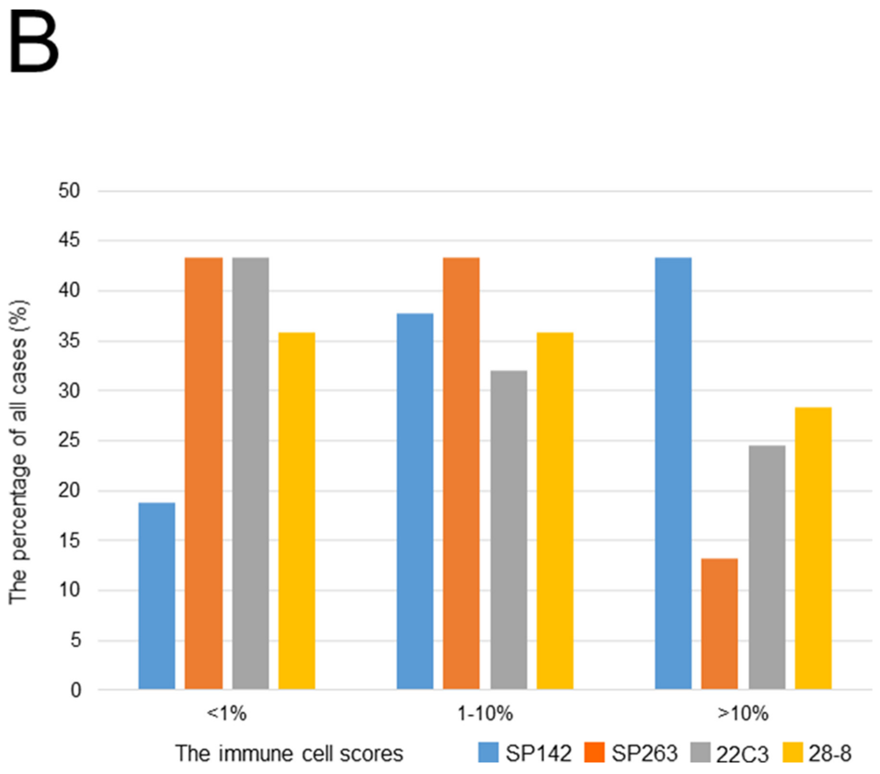

Supplementary Figure 2: (A) The percentage of cases divided by the TC scores (the ratio of positive TCs in all carcinoma cells) in thymic carcinomas for the four PD-L1 antibodies (SP142, SP263, 22C3, and 28-8). **(B)** The percentage of cases divided by the IC scores (the ratio of the area covered by stained ICs in the tumor area) in thymic carcinomas for the four PD-L1 antibodies (SP142, SP263, 22C3, and 28-8)

Supplementary Table 1: The statistical analysis of surgical cases

Factor		SP142			SP263			22C3			28-8		
		TC>1%	TC<1%	p value	TC>25%	TC<25%	p value	TC>1%	TC<1%	p value	TC>1%	TC<1%	p value
	Total cases	33	6		21	18		25	14		31	8	
Sex	Male	18	4	0.6790	12	10	1.0000	14	8	1.0000	17	5	1.0000
	Female	15	2		9	8		11	6		14	3	
Age years	≧60	17	4	0.6674	11	10	1.0000	15	6	0.3373	16	5	0.7023
	<60	16	2		10	8		10	8		15	3	
WHO stage	Stage I/II	10	2	1.0000	8	4	0.3221	10	2	0.1509	11	1	0.3938
	Stage III/IV	23	4		13	14		15	12		20	7	
Masaoka-Koga stage	Stage I/II	11	2	1.0000	9	4	0.3070	11	2	0.0826	12	1	0.2286
	Stage III/IV	22	4		12	14		14	12		19	7	
Tumor size (mm)	≧50	15	4	0.3662	11	8	0.4861	11	8	0.0466	15	4	0.1192
	<50	13	1		10	4		13	1		14	0	
Curability	Complete resection	31	6	1.0000	21	16	0.2065	24	13	1.0000	30	7	0.3725
	Imcomplete resection/biopsy	2	0		0	2		1	1		1	1	
Histological subtype	Squamous cell carcinoma	27	1	0.0041	19	9	0.0107	21	7	0.0331	24	4	0.1879
	Other type carcinoma	6	5		2	9		4	7		7	4	

Abbreviation: TC, tumor cells.

Supplementary Table 2: The results of the univariate and multivariate analyses of prognostic factors affecting the RFS in patients with thymic squamous cell carcinoma

Factor		Recurrence-free survival					
		univariate analysis			multivariate analysis		
		HR	95% CI	p value	HR	95% CI	p value
Sex	Male	0.890	0.285-2.697	0.8352			
	Female						
Age years	≧60	0.455	0.134-1.435	0.1775			
	<60						
WHO stage	Stage I/II	0.223	0.034-0.836	0.0239	0.2894	0.035-1.656	0.1720
	Stage III/IV						
Masaoka-Koga stage	Stage I/II	0.223	0.034-0.836	0.0239	NA	NA	NA
	Stage III/IV						
Tumor size (mm)	≧50	2.938	0.837-13.541	0.0943	1.4159	0.303-8.682	0.6740
	<50						
Curability	Complete resection	NA	NA	NA			
	Imcomplete resection/Biopsy						
SP142 1% cutoff		2.616	0.501-48.107	0.2971			
SP263 25% cutoff		0.499	0.164-1.565	0.2240			
22C3 1% cutoff		0.486	0.161-1.618	0.2249			
28-8 1% cutoff		1.236	0.324-8.096	0.7807			
SP142 50% cutoff		0.499	0.164-1.565	0.2240			
SP263 50% cutoff		0.492	0.110-1.617	0.2558			
22C3 50% cutoff		0.429	0.116-1.326	0.1445			
28-8 50% cutoff		0.429	0.116-1.326	0.1445			

Abbreviation: RFS, recurrence-free survival; HR, hazard ratio; CI, confidence interval.

Supplementary Table 3: Evaluation of multicollinearity in multivariate Cox regression analysis for OS

	VIF (1st step*)	VIF (2nd step)
WHO stage	1.134	1.127
SP263 25% cutoff	2.263	1.720
SP263 50% cutoff	2.612	2.184
22C3 50% cutoff	8.708	1.720
28-8 50% cutoff	8.300	Excluded

*VIF among factors whose p values were < 0.1 in univariate Cox regression analysis.
Abbreviation: OS, overall survival; VIF, variance inflation factor.

Supplementary Table 4: Evaluation of multicollinearity in multivariate Cox regression analysis for RFS

	VIF (1st step*)
WHO stage	1.458
Tumor size	1.458

*VIF among factors whose p values were < 0.1 in univariate Cox regression analysis.
Abbreviation: RFS, recurrence-free survival; VIF, variance inflation factor.
